# March of the Titans: The Locomotor Capabilities of Sauropod Dinosaurs

**DOI:** 10.1371/journal.pone.0078733

**Published:** 2013-10-30

**Authors:** William Irvin Sellers, Lee Margetts, Rodolfo Aníbal Coria, Phillip Lars Manning

**Affiliations:** 1 Faculty of Life Sciences, University of Manchester, Manchester, Greater Manchester, United Kingdom; 2 IT Services for Research, University of Manchester, Manchester, Greater Manchester, United Kingdom; 3 CONICET - Universidad Nacional de Río Negro - Subsecretaría de Cultura de Neuquén, Museo Carmen Funes, Plaza Huincul, Neuquén, Argentina; 4 School of Earth, Atmospheric & Environmental Sciences, University of Manchester, Manchester, Greater Manchester, United Kingdom; University of Utah, United States of America

## Introduction

In organismal biology, whether the focus is comparative anatomy, functional morphology or evolution, the body mass of an organism is perhaps the most important individual factor [1]–[4]. This is especially true in biomechanics. Here size has a pervasive influence on the performance of animals in their environments, and represents a primary determinant of how animals forage, fight, flee and interact [5]. This applies particularly to terrestrial vertebrates whose limbs must support the body mass against gravity and exert the necessary forces to locomote through an environment. Considering the limited range of biomaterials and their uniform physical properties [6] the size range of extant terrestrial vertebrates is impressive: adult pygmy shrews typically weigh about 0.002 kg while elephants are known to reach masses of 7000 kg [7], [8]. However, modern day giants pale into insignificance when compared to the enormous size achieved by the largest Mesozoic dinosaurs. Predatory theropod dinosaurs like *Tyrannosaurus rex* may have reached masses in excess of 10,000 kg [9], while giant sauropods are consistently estimated to have masses in the 15,000 to 40,000 kg range [10] with some perhaps reaching masses as high as 100,000 kg [11], [12].

Studies of the effects of body size on locomotor performance date back to the 1940 s and the now famous Friday Evening Discourse at the Royal Institution [13]. The two fundamental observations are (1) that muscle power is more or less proportion to muscle mass, and therefore power limited activities such as jumping should be expected to be mass independent, and (2) that muscle force is more or less proportional to muscle area which scales as mass^(2/3)^ so that force limited activities such as standing should be expected to become harder as mass increases. These are, of course, first approximations and most activities have a considerably more complex set of requirements. However the scaling of force with body size does mean that we would expect considerable locomotor constraints at large body mass. In terms of static forces it can be shown that both skeletal and muscular strength should scale adequately up to very large body sizes in the order of 100,000 to 1,000,000 kg [14]. However the situation for dynamic forces is considerably more complex and even among living animals we can observe locomotor kinematics changes with large body size to reduce the forces required during locomotion [15]. It is therefore clear that whilst we can get a great deal of useful information from studies of locomotion in the largest living terrestrial vertebrates (e.g. [16]–[19], we should expect the locomotor kinematics of the largest sauropods to differ from those seen in modern animals since they are potentially an order of magnitude larger, and have their own unique musculoskeletal adaptations such as air sacs and bone pneumacity [10].

Traditionally, both osteology and ichnology have been the only available tools for approaching sauropod limb kinematics [20]–[23]. Among titanosaurs, the most common information sources lie on features of their appendicular skeleton, which include the presence of a prominent olecranon in the ulna, laterally expanded preacetabular lobe of the ilium, proximal one-third of the femoral shaft deflected medially, and extremely elliptical femoral midshaft [22], [24]. These features are also useful to explain the trackways patterns of these graviportal animals. In contrast, bone scaling and biomechanical analysis shows little to distinguish sauropods from other quadrupedal dinosaurs [25]. Ichnological analysis has been used to calculate the speeds of titanosaur trackways [26], [27] but this may only encompasses a subset of possible gaits due to preservational bias [28], and is subject to a number of caveats in terms of accuracy [29].

Since we cannot assume, *a priori*, that sauropods used similar kinematic patterns to extant animals during locomotion, we need to generate a number of plausible locomotor patterns and test them for their efficacy in terms of biologically and mechanically meaningful measures such as skeleton and joint loading, metabolic energy cost, speed and acceleration. The general approach is to construct a computer simulation of sufficient biofidelity to capture the necessary mechanics of the system and to use this to test specific locomotor hypotheses. The earliest musculoskeletal models for use in reconstructing gait in vertebrate fossils date back to the pioneering work of Yamazaki et al. [30] who produced a highly sophisticated neuromusculoskeletal simulation to investigate the evolution of bipedality in humans and other primates. Since then a range of other vertebrate fossils have been simulated including hominoids [31]–[38], terror birds [39], and dinosaurs [40]–[44]. These simulations can be kinematically based where a movement pattern is provided founded on extant analogues, trackway data, or theoretically derived. The model then calculates the muscle activations needed to match the input kinematics. Alternatively the simulations can use global optimisation goals to optimise some output measure such as metabolic energy cost or speed. The advantage of this latter approach is that no assumptions need to be made about the likely kinematics and this makes it very suitable for situations where there may be no reasonable modern analogue. The disadvantage is that because the input is much less constrained, the simulation needs to try many more different possibilities whilst searching for the optimal solution and this makes the process extremely computationally intensive.

## Methods

Musculoskeletal systems in vertebrates are extremely complex and constructing a simulation with an appropriate level of realism to test its locomotor capabilities is a relatively time consuming process. The necessary stages are as follows.

### Skeletal Capture

**Figure 1:**
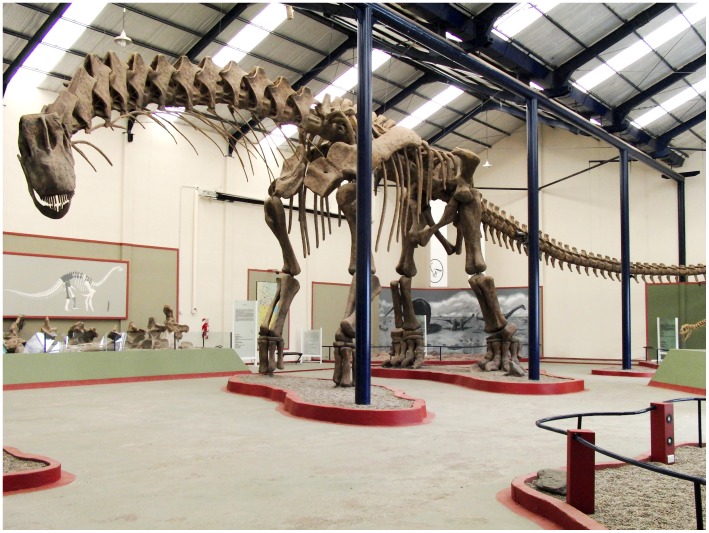
*Argentinosaurus huinculensis* reconstruction at Museo Municipal Carmen Funes, Plaza Huincul, Neuquén, Argentina.

**Figure 2:**
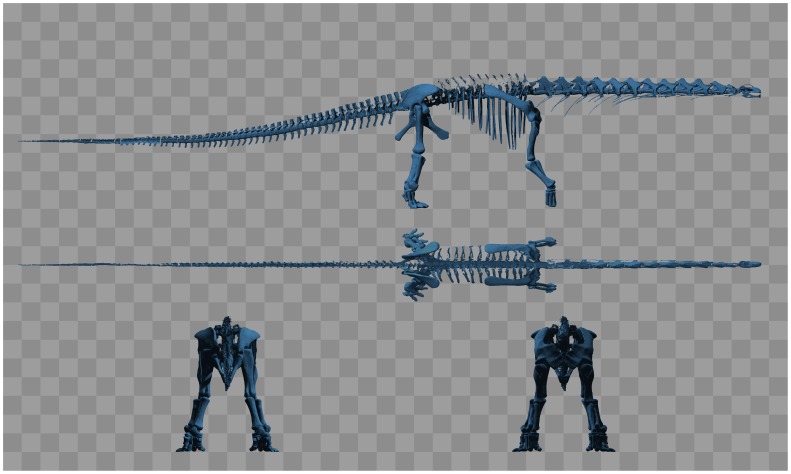
Multiple orthographic views of the digitised skeleton created using the POVRAY ray-tracer (www.povray.org). The background pattern consists of 1

The initial stage in building the simulation is construction an appropriate musculoskeletal model. The first step is to acquire a digital model of the skeleton of the target species. In this case, our aim is to explore the locomotor capabilities of the largest of the sauropod dinosaurs and we chose the to use *Argentinosaurus huinculensis*, as reconstructed by the Museo Municipal Carmen Funes, Plaza Huincul, Argentina, which also houses the original fossil material. Permission was granted by Museo Municipal Carmen Funes, Plaza Huincul, Argentina to scan their reconstruction. The reconstruction was performed in-house at the museum. This reconstruction is shown in Figure 1. It is 39.7 m long and stands 7.3 m high at the shoulder. The reconstruction is based on rather fragmentary material [45] but includes well preserved fibula and vertebral elements that have allowed mass estimates to be obtained of between 60 and 88 tonnes depending on the regression equation used [46]. The reconstruction was scanned using a Z+F Imager 5006i LiDAR scanner from multiple locations in the gallery. The individual scans were aligned by Z+F Germany, using the multiple printed targets placed around the gallery as automatically detectable shared reference points. The tail, torso, neck and head and the individual limb bones and girdles were segmented out and decimated using of Geomagic Studio (www.geomagic.com) and the resultant 3D objects posed using 3DS Max (www.autodesk.com). The quality of the scan is variable due to limitations on where the scanner could be placed. Therefore limb bones on the side that had been better scanned were mirrored to produce a completely symmetrical model and the torso was moved slightly so that its centre of mass was exactly in the midline. This produced the reference pose illustrated in Figure 2. It was not possible to raise the scanner above floor level so the quality of the scan for dorsal elements such as neural spines is relatively poor. However the limb bones and girdles are well digitised and these are the most important in terms of subsequent modelling steps.

### Segmental Mass Properties

**Figure 3:**
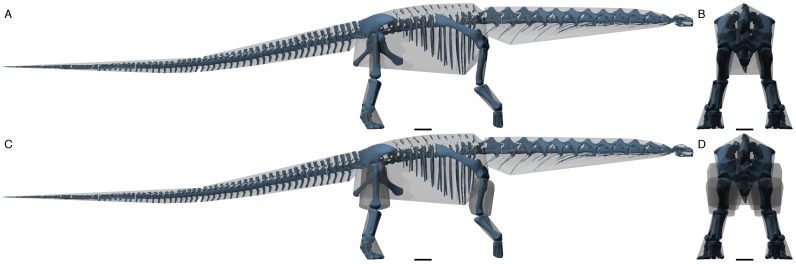
Orthographic views of the hulled segments created using the POVRAY ray-tracer (www.povray.org). A, side, and B, front view of the unscaled hull model. C, side, and D, front view of the scaled model with extra mass in the thigh and forearm segments.

Once the skeleton has been captured it is necessary to define the body segments that are used in the simulation. In common with nearly all locomotor analysis, the body is treated as a series of rigid, linked segments [47]. As in all modelling exercises it is necessary to decide on the level of complexity that is going to be used. It is perfectly possible to model every single bone as a separate segment but doing so greatly increases the calculation time for the simulation and having a large mass difference between body elements tends to cause numerical instability. For the sauropod model, 3 segments were defined for each limb representing the stylopodium, zeugopodium and autopodium. The head, neck, torso and tail were considered a single combined segment. Each segment is a six degree of freedom rigid element that has a position and orientation as well as a mass and inertial tensor. In the reference pose, the position is defined as the position of the centre of mass of the segment, and the orientation is set to a rotation of zero, with the inertial tensor calculated at this orientation. In the palaeontological literature there are two approaches for generating mass properties. Firstly these can be scaled from experimentally derived data of similarly shaped modern species and this is probably the commonest approach among hominoid workers (e.g. [32], [35]) with reference data from humans [48], [49] or chimpanzees [50]. Secondly these can be obtained from volumetric models of the target animal [51]–[53]. The modern locomotor analogues for dinosaurs have very different body shapes so the scaling approach is probably less useful than the volumetric approach. However whilst these are based on external body measurements when used with living animals, for fossil animals these soft-tissue measurements cannot be measured directly. This leads to an undesirable subjective element to these reconstructions and in an attempt to improve on this we have developed an objective technique based on convex hulling [54]. In its original form, this technique produced a mathematically unique minimum wrap around the individual skeletal components to estimate body mass. However since these are simply closed 3D shapes, all the other mass properties can also be calculated. The only difficulty is that our previous analysis found that approximately 20% of the mass was lost in the minimal wrap and this needs to be recovered. Figure 3AB shows the results of convex hulling the skeletal elements. The main place where the segments are clearly far too small is the thigh and upper arm and so the missing mass was added to these segments by using an appropriate scale factors orthogonal to the long axis of the bone. Figure 3CD shows the effects of this scaling. This choice of where to put the extra mass is somewhat arbitrary but it is believed that at low speeds, the choice of mass properties in the limbs is relatively unimportant [55]. The calculated mass properties for each segment in the reference pose are shown in Table 1. The total calculated body mass for the reconstruction using convex hulling approach [54] is 83,230 kg which is within the range previously predicted for this species [46] and certainly helps us have confidence in the reconstruction. However it must be remembered that these values are necessarily estimates. We do know how much soft tissue was associated with the skeletal segments and these estimates are means based on a limited dataset of modern animals. However we also know that the choice of mass parameters has relatively little effect on experimental [55] or simulation outcomes [33], [56].

**Table 1:** Segmental mass properties of the model as posed in the reference position.

	Position of CM (m)			SegmentMass (kg)	Moments of Inertia (kg.m^2^)			Products of Inertia (kg.m^2^)		
	x	y	z		Ixx	Iyy	Izz	Ixy	Ixz	Iyz
Left Arm	3.397	1.270	3.641	2.879E+03	1.519E+03	1.281E+03	8.795E+02	1.182E+02	−6.649E+01	−2.591E+01
Left Foot	−2.977	1.913	0.589	9.761E+02	2.199E+02	1.966E+02	1.908E+02	1.485E+01	4.443E+01	−1.427E+00
Left Forearm	3.779	1.621	1.835	4.282E+02	7.766E+01	1.251E+02	6.805E+01	−9.099E+00	4.994E+01	9.129E+00
Left Hand	4.320	1.753	0.610	1.957E+02	1.774E+01	1.565E+01	9.555E+00	1.221E+00	8.048E−01	8.834E−02
Left Shank	−2.946	1.493	2.067	6.202E+02	1.636E+02	1.613E+02	6.334E+01	1.053E+00	−3.237E+01	2.818E+01
Left Thigh	−2.763	0.998	4.219	5.387E+03	4.513E+03	3.536E+03	2.659E+03	−3.189E+02	5.098E+01	3.073E+02
Right Arm	3.397	−1.270	3.641	2.879E+03	1.519E+03	1.281E+03	8.795E+02	−1.182E+02	−6.649E+01	2.591E+01
Right Foot	−2.977	−1.913	0.589	9.761E+02	2.199E+02	1.966E+02	1.908E+02	−1.485E+01	4.443E+01	1.427E+00
Right Forearm	3.779	−1.621	1.835	4.282E+02	7.766E+01	1.251E+02	6.805E+01	9.099E+00	4.994E+01	−9.129E+00
Right Hand	4.320	−1.753	0.610	1.957E+02	1.774E+01	1.565E+01	9.555E+00	−1.221E+00	8.048E−01	−8.834E−02
Right Shank	−2.946	−1.493	2.067	6.202E+02	1.636E+02	1.613E+02	6.334E+01	−1.053E+00	−3.237E+01	−2.818E+01
Right Thigh	−2.763	−0.998	4.219	5.387E+03	4.513E+03	3.536E+03	2.659E+03	3.189E+02	5.098E+01	−3.073E+02
Trunk	0.454	0.000	5.256	6.226E+04	8.831E+04	1.281E+06	1.257E+06	2.209E+03	−8.752E+04	5.735E+02

### Muscle and Joint Locations

**Figure 4:**
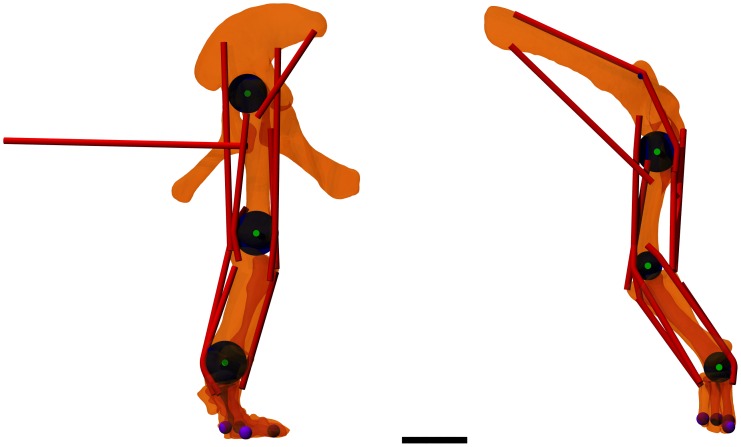
Orthographic views of the limb bones, muscle paths, wrapping cylinders, joint axes and contact points used in the model. The scale bar is 1-tracer (www.povray.org).

From the reference skeleton it is now possible to define the joints and muscle paths, although there will always be ambiguities in specific cases. As with the choice of segments, it is necessary to simplify these to prevent undue model complexity. The joints were therefore all considered to be hinge joints operating in various parasagittal planes (i.e. with hinge axes directed laterally), with the joint centre measured from the skeleton. This is probably reasonably accurate for all the joints except the shoulder and hip joints, which should be ball-and-socket joints. However it is likely that there is very little abduction/adduction or axial rotation in normal walking so this is a reasonable approximation for a model of straight line walking and greatly simplifies the control processes. The joints chosen are listed in Table 2. It is also necessary to define contact points on the skeleton which are simply the parts of the feet that make contact with the ground. The foot contact points chosen are listed in Table 3. We also define contact points on the head and the tail but these are simply used to abort the model if the simulation falls over. Muscles are another area where simplification is necessary. It is actually very straightforward to simulate a large number of muscles and this causes very few problems, and relatively little simulation computational cost. However, each muscle needs to have its activation level controlled and therefore each additional muscle increases the dimensionality of the optimal control search space. This causes a huge additional cost in terms of search and it is therefore important to have as few functional muscles as possible. Since we also have the problem that we do not know the sizes of the individual muscles even if we can infer their probably identity using an extant phylogenetic bracket [57] it makes sense to reduce the model’s complexity by using a more idealised set of muscles that represent the functional actions that are likely to be available. These muscles can be defined with arbitrary paths and moment arms as long as they produce equivalent actions to anatomical muscles. The muscles chosen are listed in Table 4, including their origin and insertion points, and illustrated in Figure 4. Most muscles are not implemented as simple point-to-point muscles. This is because they need to wrap around bones to maintain their moment arms throughout the range of movement. This effect can be achieved using multiple via points but this approach often leads to unrealistic muscle paths at the extremes of joint action. It is also possible to define the muscle as a chain of linked segments and to calculate how these would slide over the bone morphology (and even other muscles). This is very computationally expensive and can cause numerical instability issues. Instead we define cylinders or pairs of parallel cylinders that allow a wrapping path to be calculated as needed with relatively minimal cost. The radius of the cylinder is chosen to match the effective moment arm of the muscle as it wraps around the condyles of the long bones.

**Table 2:** Reference positions of the joint centres in the model.

	X (m)	Y (m)	Z (m)
Right Hip	−2.866	−0.655	5.309
Right Knee	−2.732	−1.223	3.169
Right Ankle	−3.211	−1.708	1.186
Right Shoulder	3.409	−1.217	4.417
Right Elbow	3.268	−1.347	2.670
Right Wrist	4.359	−1.610	1.116
Left Hip	−2.866	0.655	5.309
Left Knee	−2.732	1.223	3.169
Left Ankle	−3.211	1.708	1.186
Left Shoulder	3.409	1.217	4.417
Left Elbow	3.268	1.347	2.670
Left Wrist	4.359	1.610	1.116

**Table 3:** The locations of the contact spheres attached to the autopodia of the model.

Contact Name	X (m)	Y (m)	Z (m)	Radius (m)
Left Foot 1	−3.206	2.294	0.194	0.1
Left Foot 2	−2.991	1.454	0.199	0.1
Left Foot 3	−2.895	2.734	0.112	0.1
Left Foot 4	−2.466	1.304	0.141	0.1
Left Hand 1	4.111	1.920	0.327	0.1
Left Hand 2	4.321	1.495	0.318	0.1
Left Hand 3	4.505	1.835	0.205	0.1
Left Hand 4	4.502	1.605	0.295	0.1
Right Foot 1	−3.206	−2.294	0.194	0.1
Right Foot 2	−2.991	−1.454	0.199	0.1
Right Foot 3	−2.895	−2.734	0.112	0.1
Right Foot 4	−2.466	−1.304	0.141	0.1
Right Hand 1	4.111	−1.920	0.327	0.1
Right Hand 2	4.321	−1.495	0.318	0.1
Right Hand 3	4.505	−1.835	0.205	0.1
Right Hand 4	4.502	−1.605	0.295	0.1

**Table 4:** Origin and insertion positions of the muscles used in the model in the reference pose.

	Origin			Insertion			Radius 1	Radius 2
	X (m)	Y (m)	Z (m)	X (m)	Y (m)	Z (m)	(m)	(m)
Left Ankle Ext	−3.059	1.359	2.652	−3.292	1.907	0.703	0.336	
Left Ankle Ext Knee Flex	−2.883	1.037	3.556	−3.373	1.637	0.701	0.344	0.336
Left Ankle Flex	−2.431	1.327	2.573	−2.954	1.734	0.788	0.260	
Left Elbow Ext	3.273	1.383	4.289	2.948	1.381	2.302	0.219	
Left Elbow Ext Wrist Flex	3.058	1.499	3.059	4.068	2.093	0.877	0.219	0.236
Left Elbow Flex	3.669	1.077	4.174	3.644	1.510	2.604	0.223	
Left Elbow Flex Wrist Ext	3.253	1.491	2.989	4.568	1.816	0.871	0.223	0.232
Left Hip Ext	−6.594	0.055	4.586	−2.871	1.127	4.503		
Left Hip Ext Knee Flex	−3.229	0.754	6.092	−3.129	1.219	2.900	0.273	0.344
Left Hip Flex	−1.838	1.735	6.267	−2.714	1.414	4.946	0.302	
Left Hip Flex Knee Ext	−2.400	1.416	6.007	−2.523	1.654	2.438	0.302	0.288
Left Knee Ext	−2.509	1.003	4.770	−2.424	1.281	2.625	0.288	
Left Knee Flex	−2.878	1.134	5.000	−2.999	1.385	2.746	0.344	
Left Shoulder Ext	1.219	1.101	6.527	3.689	1.398	4.109	0.050	0.309
Left Shoulder Ext Elbow Flex	3.812	0.135	4.750	3.673	1.484	2.590	0.309	0.223
Left Shoulder Flex	1.161	1.588	6.046	3.337	1.587	3.943	0.315	
Left Shoulder Flex Elbow Ext	3.138	1.411	4.971	3.155	1.564	2.131	0.315	0.219
Left Wrist Ext	3.772	1.508	2.400	4.560	1.610	0.850	0.232	
Left Wrist Flex	3.115	1.720	2.174	4.085	1.904	0.831	0.236	
Right Ankle Ext	−3.059	−1.359	2.652	−3.292	−1.907	0.703	0.336	
Right Ankle Ext Knee Flex	−2.883	−1.037	3.556	−3.373	−1.637	0.701	0.344	0.336
Right Ankle Flex	−2.431	−1.327	2.573	−2.954	−1.734	0.788	0.260	
Right Elbow Ext	3.273	−1.383	4.289	2.948	−1.381	2.302	0.219	
Right Elbow Ext Wrist Flex	3.058	−1.499	3.059	4.068	−2.093	0.877	0.219	0.236
Right Elbow Flex	3.669	−1.077	4.174	3.644	−1.510	2.604	0.223	
Right Elbow Flex Wrist Ext	3.253	−1.491	2.989	4.568	−1.816	0.871	0.223	0.232
Right Hip Ext	−6.594	−0.055	4.586	−2.871	−1.127	4.503		
Right Hip Ext Knee Flex	−3.229	−0.754	6.092	−3.129	−1.219	2.900	0.273	0.344
Right Hip Flex	−1.838	−1.735	6.267	−2.714	−1.414	4.946	0.302	
Right Hip Flex Knee Ext	−2.400	−1.416	6.007	−2.523	−1.654	2.438	0.302	0.288
Right Knee Ext	−2.509	−1.003	4.770	−2.424	−1.281	2.625	0.288	
Right Knee Flex	−2.878	−1.134	5.000	−2.999	−1.385	2.746	0.344	
Right Shoulder Ext	1.219	−1.101	6.527	3.689	−1.398	4.109	0.050	0.309
Right Shoulder Ext Elbow Flex	3.812	−0.135	4.750	3.673	−1.484	2.590	0.309	0.223
Right Shoulder Flex	1.161	−1.588	6.046	3.337	−1.587	3.943	0.315	
Right Shoulder Flex Elbow Ext	3.138	−1.411	4.971	3.155	−1.564	2.131	0.315	0.219
Right Wrist Ext	3.772	−1.508	2.400	4.560	−1.610	0.850	0.232	
Right Wrist Flex	3.115	−1.720	2.174	4.085	−1.904	0.831	0.236	

Radius 1 is the proximal cylinder radius and radius 2 is the distal cylinder radius for one and two cylinder wrapping muscles.

### Muscle Properties

**Figure 5:**
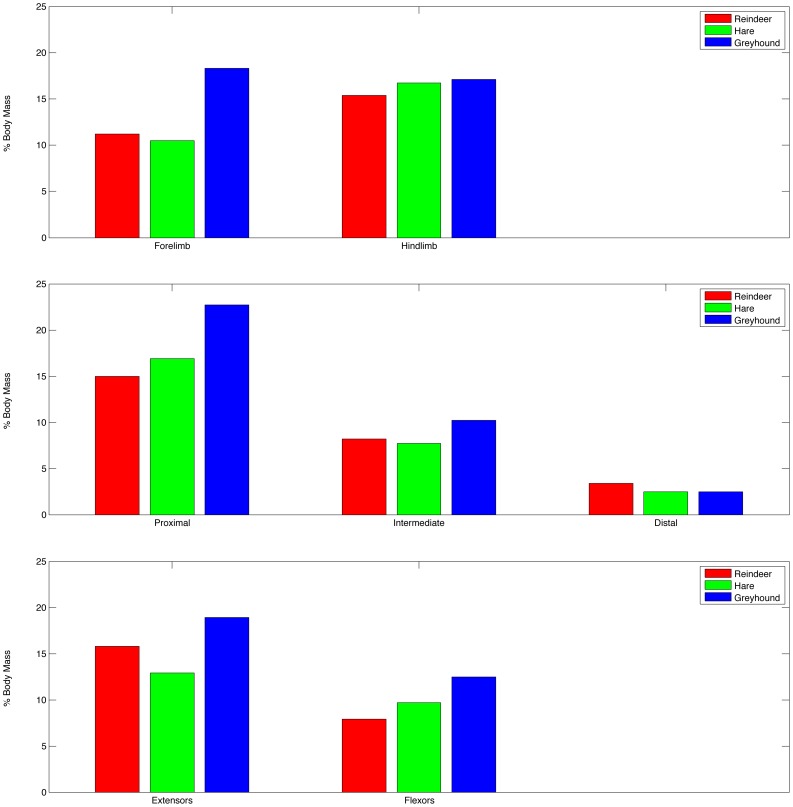
Charts showing the distribution of muscle mass in three species of cursorial quadruped. Data from Wareing et

As has been shown on several occasions [43], [56], [58], the most important property to estimate correctly in locomotor simulations is muscle mass. This is because the power available is proportional to muscle mass, and the force available, which is proportional to muscle area, is therefore proportional to the (muscle mass/muscle fibre length). Limb muscle mass as a fraction of total body mass is known for a number of animals and it is usually assumed that a value of 50% is an absolute maximum [58] and with values of 25 to 35% found more typically [59]. From the limited current data an approximate partitioning can be estimated with ∼60% of the muscle found around proximal joints, ∼30% around the intermediate, and ∼10% around the distal joints. Similarly muscle is split approximately ∼60% extensors to ∼40% flexors and ∼45% forelimb to ∼55% hindlimb [59]. Comparative data for greyhound, hare and reindeer are shown in Figure 5 and it can be seen that there is a relatively consistent pattern even for quadrupeds of different sizes and locomotor specialisations. Knowing these patterns it is therefore possible to calculate the masses of the individual muscles in the model based on their actions. This procedure works with any number of muscles as long as we assume that the mass is distributed evenly. Multiple joint muscles are simply divided among their multiple actions. To do this we need to use the model parameters listed in Table 5. Muscle density used is 1056 kg m^−3^ [60]. Force per unit area was chosen to be 300,000 Nm^−2^ [61] but there are other values in the literature: Umberger et al [62] uses 250,000 Nm^−2^, Alexander [63] reports an *in vitro* maximum value of 360,000 Nm^−2^ for frog and 330,000 Nm^−2^ for cat for parallel fibred leg muscles. Zheng et al. [64] recommend a value of 400,000 Nm^−2^ for human quadriceps, and Pierrynowski [65] suggests 350,000 Nm^−2^. There is a similarly large range for maximum contraction speed. Winter [47] suggest values from 6 to 10 times the muscle’s resting length per second for humans. This value is clearly highly dependent both on the fibre type composition of the muscle and on the temperature. Westneat [66] reports a range of values for fish from 3 to 10 s^−1^ for different fibre types and Umberger et al [62] recommends values of 12 s^−1^ for fast twitch and 4.8 s^−1^ for slow twitch. A value of 8.4 s^−1^ was chosen to represent a mixed fibred muscle. However it should be noted that there is data to suggest that this value reduces with body size [67] although there is very little data for large bodied animals and there is considerable scatter. The activation K value used is the recommended value for the muscle contraction and energetics model used [68].

**Table 5:** Fixed modelling parameters. For sources see the main text.

Model Parameter	Value
Body Mass (kg)	83,230.29
Limb Muscle Proportion	0.35
Extension to Fibre Length Ratio	0.50
Muscle Density (kg.m^−3^)	1056.00
Extensors Proportion	0.60
Flexors Proportion	0.40
Proximal Joints Proportion	0.60
Intermediate Joints Proportion	0.30
Distal Joints Proportion	0.10
Forelimb Proportion	0.45
Hindlimb Proportion	0.55
Muscle Force per Unit Area (N.m^−2^)	300,000
Activation K	0.17
VMaxFactor (s^−1^)	8.4

Muscle maximum contractile force is determined by its physiological cross section area, which is calculated by dividing the muscle volume (obtained by dividing the mass by the muscle density) by the mean fibre length [47]. Unfortunately muscle fibre length is problematic to estimate. It is usually estimated by scaling from related species. This scaling can work well if there is a good modern analogue as is probably the case for early hominin musculoskeletal models [34], [35], but is considerably less reliable for morphologically more distinct species such as dinosaurs [43], [61]. This is particularly problematic if muscles with a similar action are being combined together to provide a more abstract joint driver since in that case there is no single muscle that can be used as a homologous reference. However there is a possible solution to this difficulty that can be derived from what we know about how vertebrate muscle contracts. Muscle can only generate force from approximately 60% of its resting length to about 160% [69]. Since the force follows an inverted U shaped curve we would expect most muscles to operate well within these limits in normal use, and since muscle physiology appears to be well conserved among the vertebrates, that this useful fraction of muscle length to be similar for different species. The length a muscle shortens depends on the change in angle at the joint multiplied by the moment arm [70]. So if we know the likely range of motion at a joint and the moment arm then we can predict the likely change in muscle length, and hence predict the muscle fibre length.

To test this prediction that vertebrate skeletal muscles exhibit a preferred length change, a literature survey was performed to identify suitable experimental data. What was required were studies that reported muscle fibre length and where length change could be calculated from moment arm and range of motion data. Since many muscle show changes in moment arm with joint angle this restricted studies to those where moment arm was measured over a range of joint angles. It was also decided that only studies that reported a reasonably large number of muscles should be included otherwise there would be bias associated with large numbers of studies on a relatively few specific muscles. There were relatively few suitable studies found, and of these several were of closely related primate species (hominoids including humans) and it was felt that including all these would produce a taxonomic bias. In the end the following species were chosen: chimpanzees [71], greyhound [72], [73], ostrich [74], [75] and horse [76]. For the chimpanzee, ostrich and horse the literature gave the best-fit polynomials for the tendon travel during joint rotation so that the length change of the muscle could be calculated directly. For the greyhound, the moment arm data was integrated over the range of angles presented to calculate length change. The chimpanzee and greyhound datasets included both fore- and hindlimbs whereas the ostrich and horse were hindlimb only. Ideally for this study the joint range of motion should match that seen *in vivo* for a range of movements. This is difficult to duplicate in cadaver studies since dead bodies tend to stiffen up which can restrict movement. Conversely as muscles are dissected away the joints become more mobile and this can lead to excessive movements at joints. In the case of the ostrich the joints were only moved through the range of movement associated with running and particularly for the hip and knee this was felt to be rather restricted. The analysis was repeated using a nominal, much larger range of movement for the ostrich data but this had no effect on the results and the conclusions remained unaltered so only the data as calculated directly from the paper is reported here.

**Figure 6:**
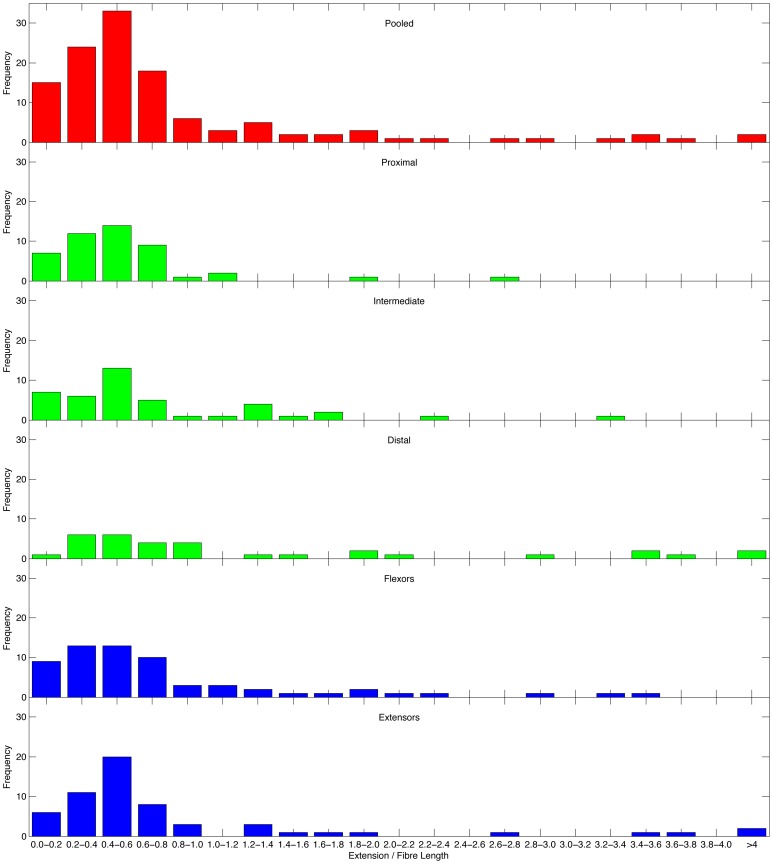
Charts showing the frequency distributions of the (extension/fibre length) ratio for a variety of muscles and vertebrate species.

Figure 6 shows the (extension/fibre length) ratios for the 121 muscles assessed subdivided by action and location. The modal value in the pooled case is 0.4–0.6, and only in two of the subdivided cases is the mode less clearly defined (0.2–0.6 in both cases). This suggests that assuming that muscle extends 50% of its resting fibre length (or conversely, that the resting fibre length is double the extension distance) is a reasonable assumption for most muscles. Very low values are probably due to one of two of factors. Firstly these are muscles whose prime action is neither flexion nor extension and therefore do not change length appreciably during this movement at the joint. Secondly these are muscles that cross more than one joint but whose action is mainly over a different joint. Very high values are more interesting because muscles cannot generate active force over these large extension ratios. Again there are two possibilities. Firstly these represent muscles that do not extend over the observed *in vitro* range *in vivo*. This includes two joint muscles where the full range of movement is not possible at both joints simultaneously. The human hamstrings are a good example of this where full hip flexion is not possible if the knee is extended. Secondly these represent muscles where part of the joint movement is accommodated by tendon stretch. The crural part of the camel *m. plantaris* is perhaps the most extreme example [77].

We can thus calculate the fibre length of the muscle by calculating the length change of the muscle which is equal to the joint range of motion multiplied by the moment arm. Moment arms are not necessarily easy to obtain for extinct species since exact points of attachment can be difficult to define. Furthermore, moment arms themselves depend on the presence of other soft tissue elements and exact instantaneous joint centres which are also unknown and need to be estimated (e.g. [42]). However if we use length change to define muscle fibre length, then the choice of moment arm does not actually matter in the simulation. If we choose a small moment arm, then we get a small length change, and hence a small fibre length. Since the volume of the muscle is defined by the mass which we have calculated *a priori*, a small fibre length leads to a large physiological cross section area which allows greater force production. Since all these relationships are directly proportional, the greater force production exactly compensates for the reduced moment arm in terms of the eventual torque around the joint. The contraction velocity is similarly exactly compensated: shorter muscle fibre, slower contraction velocity, but smaller moment arm leads to faster angular velocity around the joint. This is exactly as would be predicted from simple lever theory.

The key parameter then becomes joint range of motion. However there have been very few studies that have systematically looked at joint ranges of motion, and whilst some joint limits can be identified from skeletal features, others depend on soft tissue to limit the movement and thus can not. Ren et al. [17] compared elephant joint ranges of motion to cats, dogs, and humans and contrary to expectations did not find any body size related patterns. We thus created models with a range of different joint ranges of motion based on (1) estimation of joint range of motion from the skeleton; (2) range of motion matched to the functional range of motion for an elephant; (3) range of motion based on the previous two versions but with a restricted ankle range of motion. These ranges of motion are shown in Table 6. Using each of these ranges of motion allows us to calculate the length change of the individual muscle groups using the attachment points and wrapping cylinders previously specified. The tendon length is simply chosen so that the muscle tendon unit is slack when the joint is halfway between its maximum and minimum excursion. The calculated values for the muscles under the different range of motion conditions are shown in Table 7. Again there is no good comparative data on slack lengths and it is difficult to obtain since there is appreciable post mortem shrinkage and stiffening so that measurements taken from cadavers are probably not useful. Measuring passive elastic moments [78], as has been done for human models [79], might allow this to be calculated but the data would have to be taken from anaesthetised animals which would make it much more difficult to collect.

**Table 6:** Joint ranges of motion with respect to the reference pose.

		Best Estimate ROM (°)	Elephant Functional ROM (°)	Restricted Ankle ROM (°)
Hip	Min	−20	−20	−20
	Max	70	20	40
	Range	90	40	60
Knee	Min	−105	−50	−40
	Max	15	5	20
	Range	120	55	60
Ankle	Min	−10	−10	−30
	Max	55	30	0
	Range	65	40	30
Shoulder	Min	−75	−35	−40
	Max	15	10	20
	Range	90	45	60
Elbow	Min	−35	−20	−40
	Max	90	25	20
	Range	125	45	60
Wrist	Min	−50	−70	−5
	Max	65	35	25
	Range	115	105	30

Positive values allow the distal element to move anticlockwise when viewed from the right of the body.

**Table 7:** Muscle properties for each of the joint range of motion conditions.

Joint Range of Motion	Muscle Group	Min (m)	Max (m)	Extension (m)	FL (m)	Mass (kg)	PCSA (m^2^)	Tendon Length (m)
Best Estimate	Ankle Ext	2.115	2.485	0.371	0.741	320.44	0.4095	1.559
	Ankle Ext Knee Flex	2.340	3.462	1.122	2.245	400.55	0.1690	0.656
	Ankle Flex	1.494	1.962	0.468	0.935	320.44	0.3244	0.793
	Elbow Ext	1.802	2.360	0.558	1.116	589.89	0.5004	0.965
	Elbow Ext Wrist Flex	2.104	3.189	1.086	2.171	382.34	0.1668	0.476
	Elbow Flex	1.251	1.830	0.579	1.159	393.26	0.3213	0.382
	Elbow Flex Wrist Ext	1.865	2.919	1.054	2.107	327.72	0.1473	0.285
	Hip Ext	3.611	4.631	1.020	2.040	1922.62	0.8925	2.081
	Hip Ext Knee Flex	2.419	3.656	1.238	2.476	1201.64	0.4597	0.562
	Hip Flex	1.179	1.722	0.543	1.086	1281.75	1.1174	0.364
	Hip Flex Knee Ext	2.620	4.235	1.616	3.231	1121.53	0.3287	0.196
	Knee Ext	2.076	2.688	0.612	1.225	961.31	0.7433	1.157
	Knee Flex	1.498	2.376	0.878	1.755	480.65	0.2593	0.182
	Shoulder Ext	3.727	4.211	0.484	0.968	1573.05	1.5387	3.001
	Shoulder Ext Elbow Flex	2.128	3.109	0.982	1.963	983.16	0.4743	0.655
	Shoulder Flex	2.442	3.118	0.676	1.352	1048.70	0.7347	1.429
	Shoulder Flex Elbow Ext	2.013	3.284	1.271	2.542	819.30	0.3053	0.107
	Wrist Ext	1.522	2.004	0.482	0.963	262.18	0.2577	0.800
	Wrist Flex	1.348	2.009	0.661	1.322	174.78	0.1252	0.357
Elephant Functional	Ankle Ext	2.115	2.343	0.228	0.455	320.44	0.6666	1.774
	Ankle Ext Knee Flex	2.685	3.259	0.574	1.147	400.55	0.3307	1.825
	Ankle Flex	1.692	1.962	0.269	0.538	320.44	0.5636	1.289
	Elbow Ext	1.905	2.112	0.206	0.413	589.89	1.3542	1.596
	Elbow Ext Wrist Flex	2.090	2.820	0.731	1.461	382.34	0.2478	0.994
	Elbow Flex	1.469	1.750	0.281	0.561	393.26	0.6634	1.048
	Elbow Flex Wrist Ext	2.340	2.941	0.601	1.202	327.72	0.2583	1.439
	Hip Ext	3.611	4.141	0.530	1.059	1922.62	1.7190	2.817
	Hip Ext Knee Flex	2.677	3.359	0.682	1.364	1201.64	0.8343	1.654
	Hip Flex	1.496	1.722	0.226	0.452	1281.75	2.6825	1.157
	Hip Flex Knee Ext	3.366	3.959	0.593	1.187	1121.53	0.8950	2.476
	Knee Ext	2.137	2.413	0.276	0.552	961.31	1.6492	1.723
	Knee Flex	1.947	2.316	0.369	0.739	480.65	0.6161	1.393
	Shoulder Ext	3.754	3.996	0.242	0.484	1573.05	3.0764	3.390
	Shoulder Ext Elbow Flex	2.343	2.838	0.495	0.990	983.16	0.9400	1.600
	Shoulder Flex	2.753	3.090	0.337	0.674	1048.70	1.4727	2.247
	Shoulder Flex Elbow Ext	2.521	3.008	0.487	0.974	819.30	0.7962	1.790
	Wrist Ext	1.659	2.084	0.425	0.850	262.18	0.2922	1.022
	Wrist Flex	1.273	1.879	0.606	1.213	174.78	0.1365	0.363
Restricted Ankle	Ankle Ext	2.002	2.172	0.170	0.340	320.44	0.8914	1.746
	Ankle Ext Knee Flex	2.640	3.175	0.535	1.069	400.55	0.3547	1.838
	Ankle Flex	1.904	2.055	0.151	0.301	320.44	1.0067	1.678
	Elbow Ext	1.764	2.092	0.328	0.657	589.89	0.8508	1.272
	Elbow Ext Wrist Flex	2.343	2.750	0.407	0.814	382.34	0.4445	1.732
	Elbow Flex	1.501	1.854	0.353	0.707	393.26	0.5268	0.970
	Elbow Flex Wrist Ext	2.403	2.757	0.354	0.709	327.72	0.4378	1.871
	Hip Ext	3.611	4.377	0.766	1.531	1922.62	1.1888	2.462
	Hip Ext Knee Flex	2.755	3.544	0.789	1.578	1201.64	0.7212	1.572
	Hip Flex	1.364	1.722	0.358	0.717	1281.75	1.6939	0.826
	Hip Flex Knee Ext	3.009	3.909	0.901	1.801	1121.53	0.5896	1.658
	Knee Ext	2.041	2.363	0.323	0.645	961.31	1.4103	1.557
	Knee Flex	2.027	2.406	0.379	0.759	480.65	0.6001	1.458
	Shoulder Ext	3.700	4.022	0.323	0.645	1573.05	2.3085	3.216
	Shoulder Ext Elbow Flex	2.307	2.963	0.656	1.312	983.16	0.7095	1.323
	Shoulder Flex	2.711	3.146	0.435	0.870	1048.70	1.1415	2.058
	Shoulder Flex Elbow Ext	2.343	3.044	0.701	1.401	819.30	0.5538	1.292
	Wrist Ext	1.700	1.822	0.122	0.244	262.18	1.0178	1.517
	Wrist Flex	1.632	1.825	0.193	0.386	174.78	0.4288	1.343

**Figure 7:**
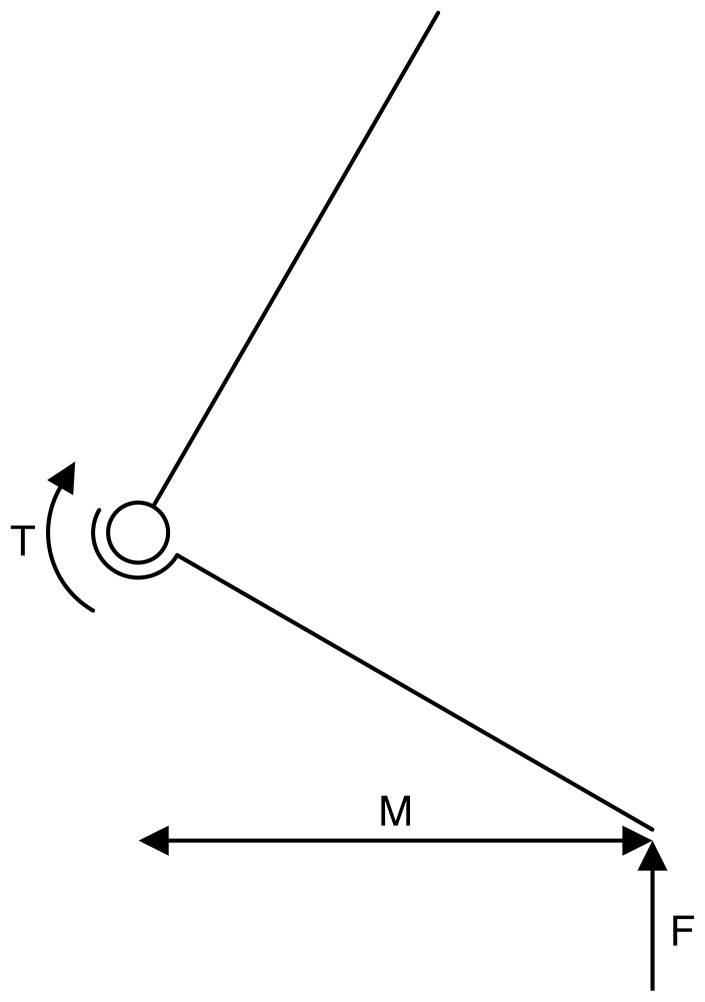
Diagram showing how the minimum ankle torque required to support an animal can be calculated.

One useful side effect of calculating muscle fibre length from joint range of motion is that you can calculate the minimum muscle mass needed for joint extensors to be able to support a particular load. This is easiest to see for the ankle or wrist but is applicable for all the joints in each limb. If we consider Figure 7 which represents the ankle joint supporting the body weight of the animal (or some fraction thereof for multi-legged animals), we can see that the torque around the ankle (T) must be equal or greater to the ground reaction force (F) multiplied by the moment arm (M). This torque is generated by the ankle extensors, and using the methodology for specifying muscle fibre length outlines above we can show that:


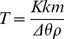
(1)

Where K is the peak force generated per unit cross section area (N.m^−2^) as specified in Table 5; k is the (extension/fibre length) ratio chosen (0.5); m is the mass of the muscle (kg); Δθ is the joint range of motion (radians); and ρ is the muscle density (Kg.m^−3^).

Since T=FM we can rearrange this equation to calculate the minimum extensor mass:


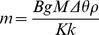
(2)

Where B is the effective body mass (kg); and g is the acceleration due to gravity (m.s^−2^). Effective body mass is the body mass that would need to be supported by this leg alone. This would be equal to the body mass for a biped but would equal 1/3 of the body mass if we assume that 3 legs were on the ground at all times.

Of these values, only Δθ is unknown for a fossil animal and thus the muscle mass is directly proportional to the joint range of motion chosen. In fact the effect of joint range of motion may be greater than that because a larger range of motion may lead to a larger horizontal moment arm too. We performed this calculation for the *Argentinosaurus* model for all the joints using the maximum possible moment arm, as calculated by the maximum horizontal distance from the foot centre of pressure to the joint centre at either full extension or full flexion, as a way of checking that the model had adequate muscle to function.

### Gait Simulation

Once all the muscle, joints, segments, and contacts have been defined it is necessary to find an appropriate activation pattern for the muscles that produces effective walking. To do this we use a feed-forward control system where a central pattern generator sends out muscle activation signals. This is a very simple approach but it is effective in a simulation environment which is entirely uniform. For these simulations we have adopted boxcar functions for the activation patterns [35]. A boxcar function is a rectangular function that has a zero value for a specified time and then a non-zero value for another specified time before falling back to zero. A boxcar function can thus be specified by 3 parameters: a delay, a width, and a height. This is a very concise way, in terms of control parameters, of specifying an activation pattern. If more precise control is required then two or more boxcar functions can be summed which rapidly allows very complex activation shapes to be generated, although single boxcar functions are the only ones that have been used in these simulations. The boxcar functions are duration normalised so that they work in a time interval from 0 to 1, and wrap around. The cycle time for all the functions is specified by a single master cycle time. The gait is assumed to be symmetrical so the left hand size drivers are identical to the right hand side drivers but are half a cycle out of phase. For these experiments the cycle phase was fixed externally. Since the model has 19 muscle groups per side, this equates to 57 unknown parameters to control the model.

We need to do two things: (1) find a good set of values for these parameters to allow high quality locomotion; (2) find a set of starting conditions that allow the simulation to work in a cyclic steady state. We do this using our now standard procedure of starting our simulant in its reference pose with all segments set at zero velocity, and using a genetic algorithm multiparameter optimisation procedure to find a pattern that maximises the forward distance moved by the model in fixed time. Once we have found a pattern that manages a good degree of forward movement, we use the segment poses and velocities from the middle of this simulation as a new set of starting conditions, and use the solution set as a best estimate solution set for a new optimisation run. This time the optimisation criteria is the maximum distance forward for a given amount of metabolic energy as calculated by the simulation. Once a good solution has been found, we repeat the process of selecting a mid-simulation set of velocities and poses, and reusing the solution set for a new optimisation run. In this way we bootstrap our start conditions, and eventually we end up converging on a largely steady state simulation that minimises the cost of locomotion since this is commonly considered the major goal of low speed locomotion [34], [35].

**Figure 8:**
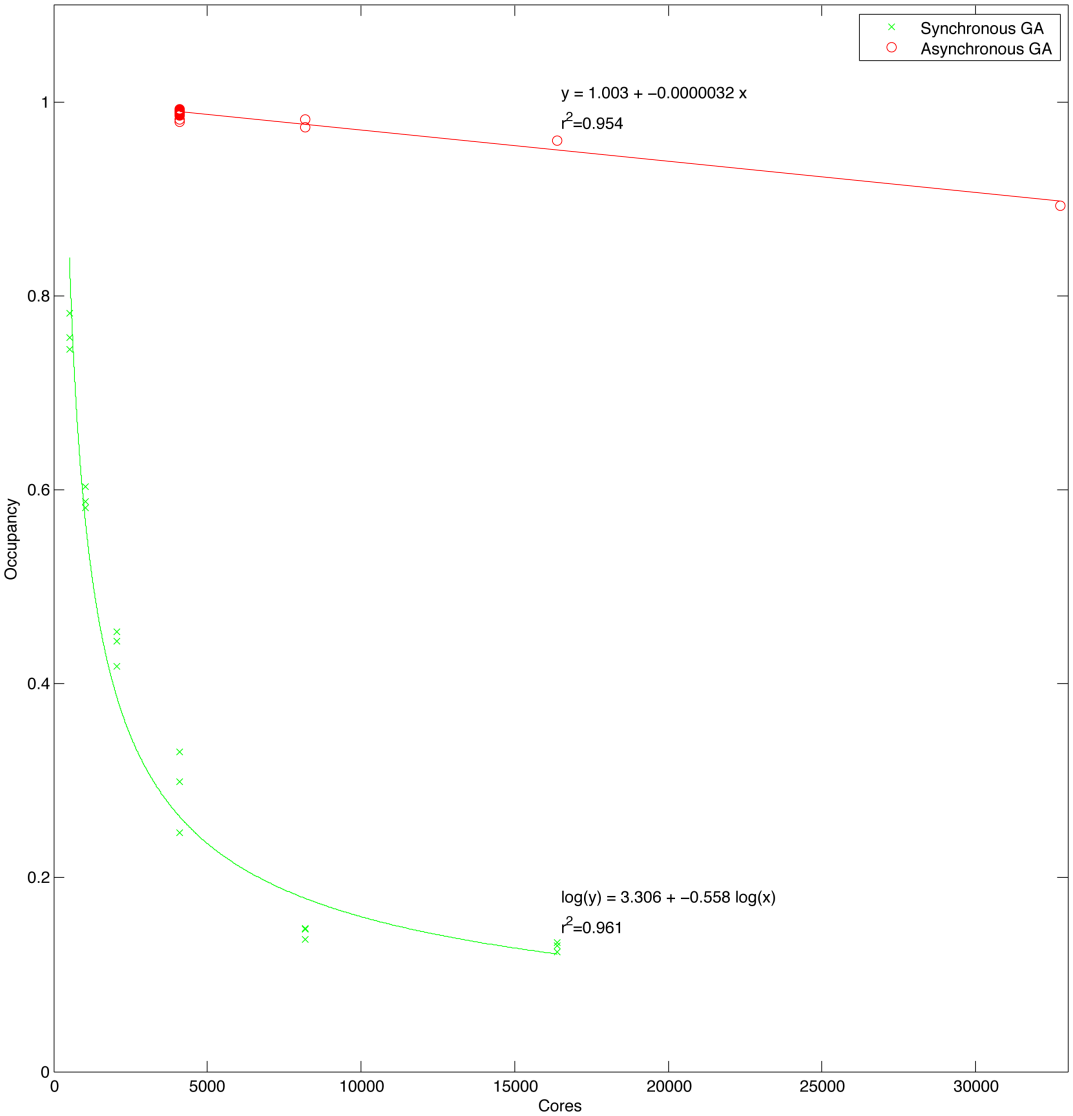
Chart showing the performance characteristics of asynchronous versus synchronous genetic algorithm implementations on varying numbers of CPU cores.

The simulation was performed using our in-house open source simulator, GaitSym. The software and the model specification files can be downloaded from www.animalsimulation.org. The simulation runs at about half real time on a modern processor, so a typical simulation run takes about 30 seconds of CPU core time. A single optimisation run requires 100,000 repeats of the simulation run, and typically 30 repeats of the bootstrap process are needed to get convergence. This equates to about 25,000 CPU core hours for each run condition tested. We had access to the HECToR, the UK National Supercomputer Service (www.hector.ac.uk) and were able to access up to 32,768 CPU cores at any one time. Our previous traditional genetic algorithm implementation [44] was very successful up to 512 cores but did not scale well for use with larger numbers of cores. Traditional genetic algorithms are highly synchronised [80], effectively because they use a seasonal breeding model. We re-implemented the algorithm using a continuous breeding and therefore asynchronous model and achieved excellent scaling up to 32,768 CPU cores (see Figure 8) which allowed us to explore considerably more options in terms of gait generation in a reasonable length of time.

## Results

**Figure 9:**
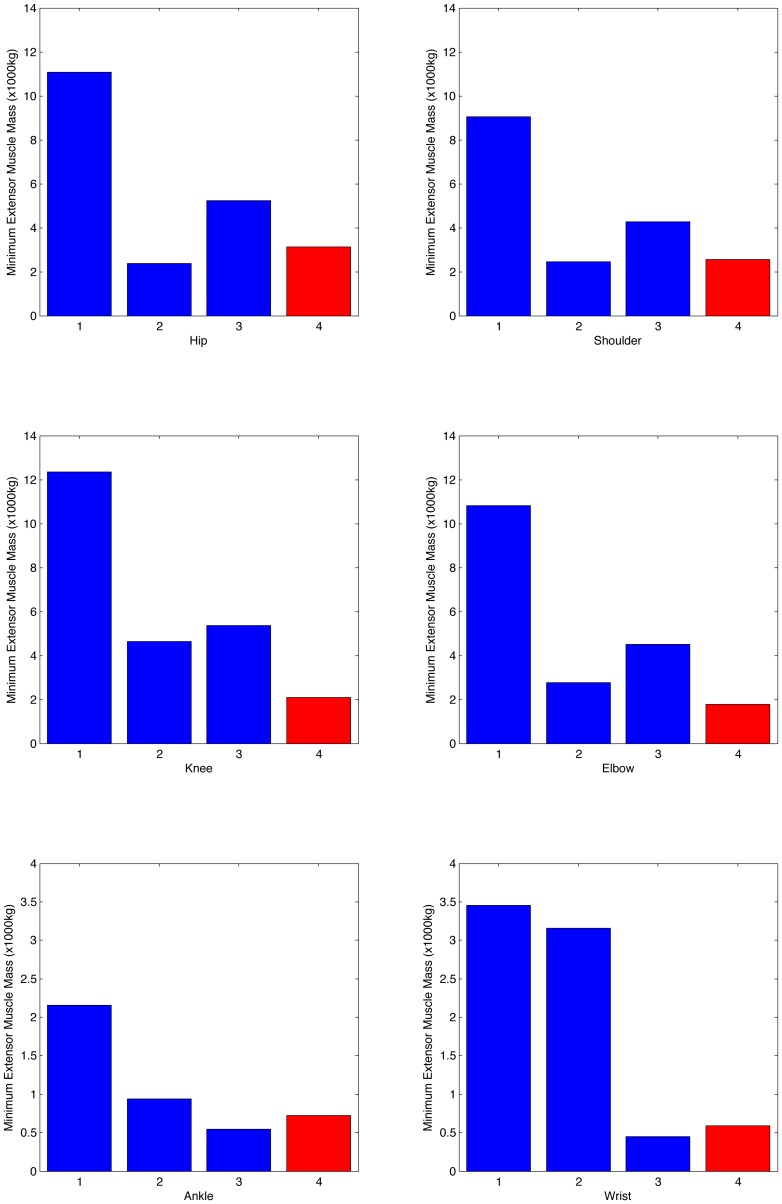
Charts showing the minimum extensor muscle mass required (1,2,3) and the muscle mass available (4) around individual joints for the different joint range of motion cases. 1, best estimate range of motion; 2, elephant functional range of motion; 3, restricted ankle range of motion; 4, muscle mass in model.

We ran the complete bootstrap process for the three joint range of motion conditions multiple times. The initial standing start was run at least 10 times in each case but only continued to the second stage if a run was found with appreciable forward movement. However the best estimate joint range of motion model was never able to generate a cyclic walking gait. The elephant functional range of motion model was able to generate cyclic gait but it did so by allowing the wrist joint to lock at a position of maximum flexion and producing a gait somewhat reminiscent of a chimpanzee knuckle walking. The restricted ankle range of motion model was able to generate good quality gait. To explore the reasons for this we calculated the minimum muscle mass required for the joint extensors for each of the cases using equation 2 and estimating the maximum possible moment arm for the available range of motion. These results are shown in Figure 9. From this it is clear why the best estimate joint range of motion model failed since there is clearly insufficient muscle mass around all of the joints to support the body with even moderate levels of joint excursion. The elephant functional range of motion model is very weak around the wrist which again matches the simulation findings where the wrist joint collapsed to full flexion. The restricted ankle range of motion model is slightly vulnerable, particularly around the knee and elbow extensors, but these values assume the maximum possible moment arm which is unlikely to be actually achieved at any point (and can to some extent be actively avoided by the global optimisation procedure), so this model is the only functional one.

**Figure 10:**
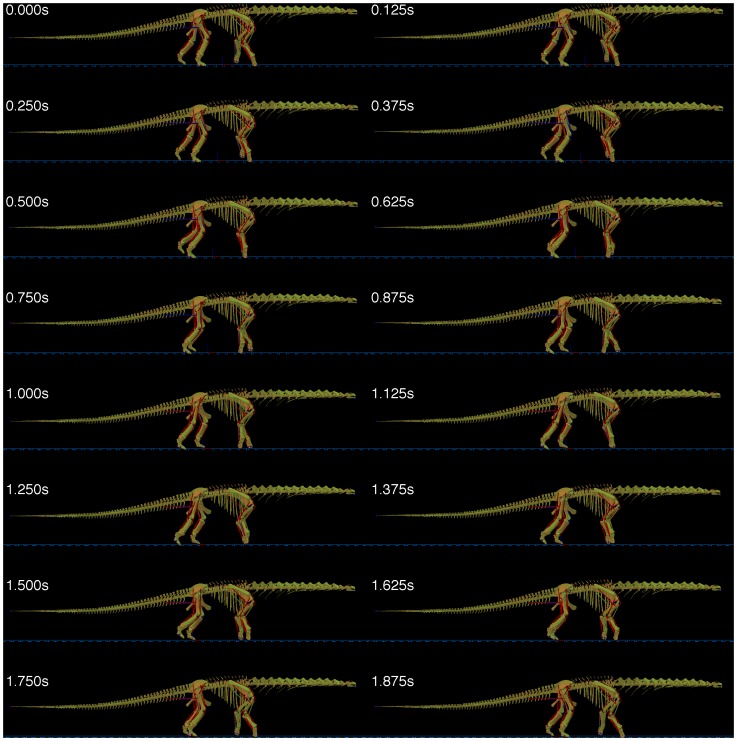
Animation frames generated by GaitSym (www.animalsimulation.org) for the 2 second gait cycle time.

**Figure 11:**
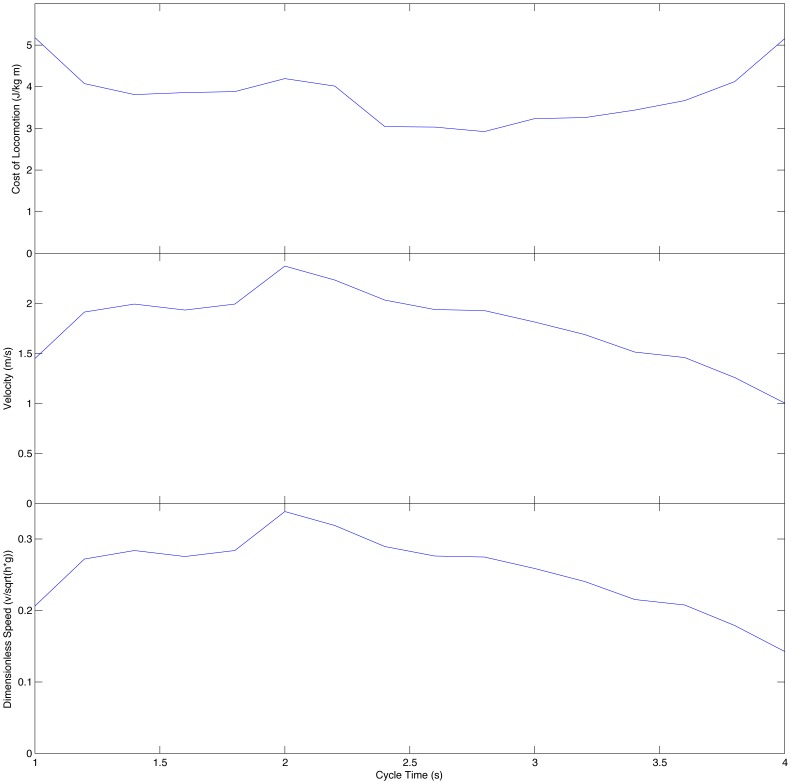
Charts showing the cost of locomotion and walking speeds for the best simulations generated with different gait cycle times.

**Figure 12:**
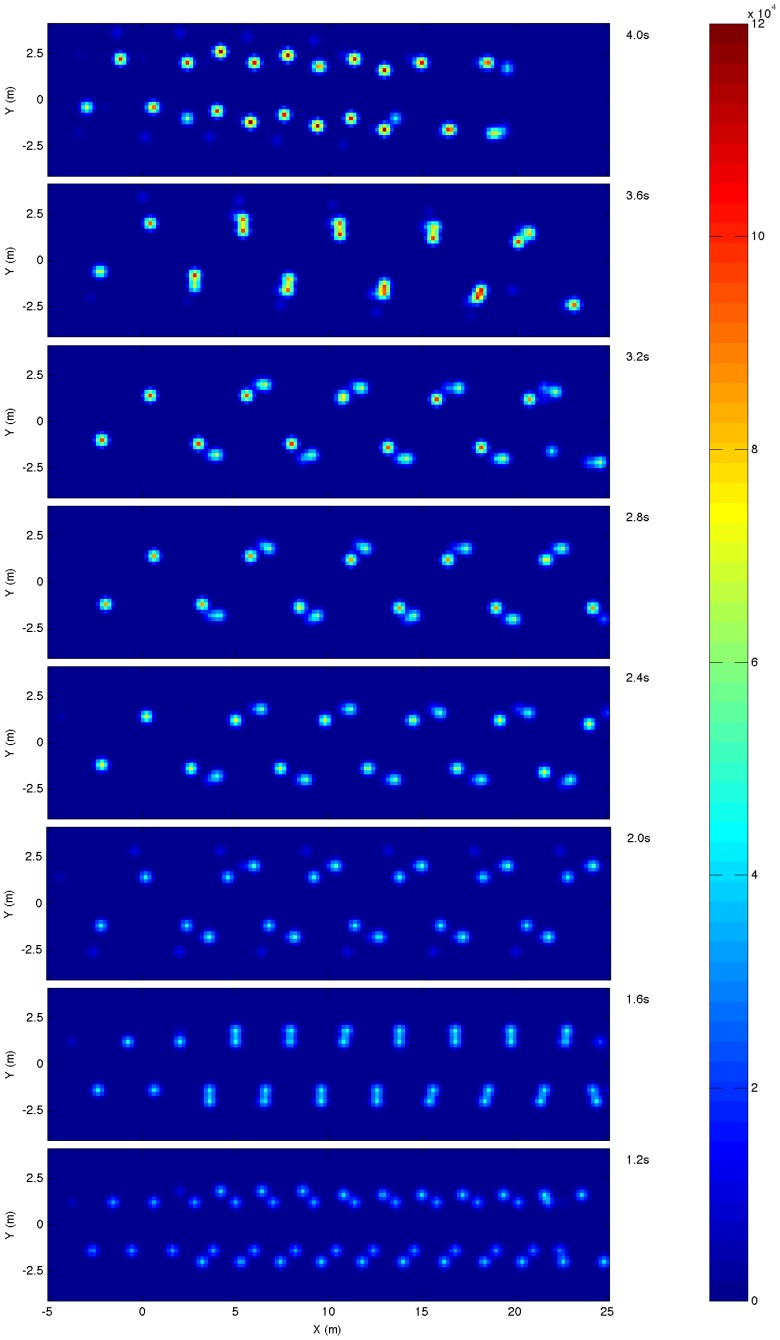
Simulated trackways generated by spatially summing the impulse between the foot contacts and the substrate.

The model was optimised to move the greatest distance forward for a fixed amount of energy and as expected this generated a slow, walking gait. This is illustrated in Figure 10 for a gait with a 2 second cycle time. A range of different gait cycle times were tried from 1.0 s to 4.0 s and the animations produced are available in the supplementary data. Because of the pendular nature of walking gaits it was expected that considerable differences would be seen in the cost of locomotion for different cycle times. As can be seen in Figure 11, the most efficient gait had a cycle frequency of 2.8 s which is relatively close to the natural frequencies of the fully extended legs (3.1 s for the forelimb and 3.7 s for the hindlimb) There was a greater difference in locomotor speed with the longer cycle times producing the fastest gaits, and the longest stride lengths, although as can be seen from the dimensionless speed (calculated as the square root of the Froude number, velocity/√(hip height × g), following Alexander [20]). For comparison, the maximum speed obtained is equivalent to a human with 0.9 m leg length walking at 1 ms^−1^ [63] which, although slower than the mean, is well within the normal range of typical walking speeds seen in free ranging humans [81]. The gait produced was typically a diagonal gait with lateral couplets [82]: foot fall sequence left hindfoot, right forefoot, right hindfoot, left forefoot; and the ipselateral forefoot and hind foot on the ground for a greater proportion of the gait cycle than the contralateral forefoot and hind foot. However the phase difference was very small and the gaits generated were very close to a pace, particularly when the cycle time was reduced. It is also useful to compare the generated gaits to trackway data. Figure 12 shows a spatial plot of the underfoot impulse which shows where individual footprints would be formed. At intermediate cycle times (2.4 to 3.2 s) these show marked similarity to standardised depictions of sauropod trackways [20].

## Discussion

The process of creating a forward dynamic simulation of *Argentinosaurus* has highlighted a number of interesting aspects of its biology. The mass estimate of 83 tonnes using the convex hull technique is relatively robust provided that the reconstruction is accurate. That it agrees broadly with estimates based on single bone allometric relationships is encouraging given the fragmentary nature of the fossil material on which it is based. Reconstructing the soft tissue parameters correctly are, of course, essential for an accurate assessment of its locomotor capabilities, and the process described here illustrates how comparative approaches can be used to find appropriate values for these parameters. However it also highlights the dearth of suitable data. Many vertebrates have been carefully dissected and their internal anatomy described in exquisite detail. Unfortunately very few vertebrates have been dissected quantitatively, and the lack of soft tissue measurements means that we do not know whether the trends that have been identified concerning muscle mass distribution are widely applicable among cursorial vertebrates. The same issues are present for joint ranges of motion: both for functional range of motion during gait and for maximum ranges of motion during other activities. The findings for muscle fibre length as a function of length change are based on a large number of muscles but relatively few (if diverse) species. Ideally this would be extended to more species but because there is a strong physiological basis for the 50% (extension/fibre length) ratio, it is likely that this finding is robust. A large data set would improve the estimate of the mode and might reveal patterns between muscles that have different primary functions. However the individual variation in this ratio is very large and deciding a specific, muscle by muscle value, for fossil animals may prove difficult.

The predictions of equation 2 fall directly from the (extension/fibre length) ratio argument and have profound effects for locomotor modelling in extinct animals. It is usually impossible, based on the fossil remains, to know how muscle is partitioned. However this equation generates a functional minimum for the muscle mass around a particular joint once a range of motion has been specified. It is particularly the case in theropod dinosaurs, with their relatively long metatarsus, that lack of sufficient ankle extensor muscle has caused problems in our earlier simulation models, and has been highlighted as a speed limiting factor in static models [42], [58]. There may be mechanical systems that can avoid this problem. Distal muscles can use parallel and serial connective tissue to increase the passive elasticity of muscles and this might allow much of the movement at the joint to be accommodated by elastic stretch rather than active contraction. There is considerable difference between the passive properties of different muscles (e.g. frog hindlimb muscles [83]) but little systematic biomechanical analysis. Similarly, clever use of multiple joint muscles with moment arms that change with joint angle may also minimise the force required at particular stages in the locomotor cycle. Alternatively, control heuristics can ensure that the load moment arm is always small when high loads are applied. In practice, it is likely that all these mechanisms come into play, but there are clear lower limits to the amount of muscle necessary to allow active force generation in situations where large ranges of joint motion are required such as standing up.

The simulation outputs reveal that it is indeed possible to generate convincing gaits using a global optimisation system provided that the fundamental mechanics of the system are gait compatible. This in itself is useful since it provides a functional bracket to soft-tissue reconstructions. However it is clear that generating efficient gait is rather difficult. The metabolic cost of locomotion has been shown to scale negatively with body mass [C=10.79 m^–0.31^ [84]]. This equation would predict a value of 0.322 J kg^−1^m^−1^ which is far lower than the 3.81 J kg^−1^m^−1^ found by the simulation. It may be that this relationship cannot be extrapolated to large body masses depending on how the mechanical cost of locomotion scales [85] since the mechanical cost per kilogram may be mass independent at approximately 1 J kg^−1^m^−1^ and the metabolic cost cannot be lower than the mechanical cost. The largest animal that we have good data for the metabolic cost of locomotion is the horse with values of about 1.5 J kg^−1^m^−1^ for a mean body mass of 515 kg. It is possible that the control pattern, based on 57 parameters, is simply not complex enough, to specify highly efficient gait. Locomotor control is certainly an area where further work is necessary, but increasing the sophistication of the control system increases the number of search parameters and this can actually lead to worse solutions being found. Systems that use incremental search are therefore potentially useful such as increasing the control complexity in subsequent repeats. Heuristics such as phase resetting may prove helpful in this context [86]. The choice of footfall pattern selected by the model is interesting because the model is free to choose footfall patterns, and there are considerable footfall pattern differences found among living species [87]. However it is clear from other work on simulation of quadrupedal gait [88] that a considerable number of repeats need to be performed before conclusions about gait selection can be made. The gaits generated are also somewhat slow but this may be a function of the relatively minimal muscle availability, or perhaps also due to the lack of elastic support structures which would stiffen the limbs and increase elastic recoil. It is clear that such passive structures, such as the stay apparatus in the horse [89], are essential for effective quadrupedal locomotion and we would predict that such would be found in sauropod dinosaurs.

There are a number of areas where the model needs to be improved. There is a great shortage of comparative neontological data and this needs to be collected to improve any soft tissue reconstruction. The model has limited biorealism at present, and future models should incorporate a full myological reconstruction. In addition spinal mobility, particularly at the neck and tail, should also be investigated. Similarly, increased complexity in the control system, particularly feedback from skeletal loading, should be incorporated. The model relies heavily on the full body skeletal reconstruction and more work needs to be done on other, more complete sauropod specimens to confirm any findings. Finally the model should be validated using a Monte Carlo sensitivity analysis [90] to investigate which parameters have the greatest effect on the model’s predictions and how these individual parameters might interact.

## Conclusions

Forward dynamic simulations shows that an 83 tonne sauropod is mechanically competent at slow speed locomotion. However it is clear that this is approaching a functional limit and that restricting the joint ranges of motion is necessary for a model without hypothetical passive support structures. Much larger terrestrial vertebrates may be possible but would probably require significant remodelling of the body shape, or significant behavioural change, to prevent joint collapse due to insufficient muscle.

## Supporting Information

Movie S1 Lateral view movie file generated from the best example using a 1.0 s gait cycle time.
(MPG)

Movie S2 Lateral view movie file generated from the best example using a 1.2 s gait cycle time.
(MPG)

Movie S3 Lateral view movie file generated from the best example using a 1.4 s gait cycle time.
(MPG)

Movie S4 Lateral view movie file generated from the best example using a 1.6 s gait cycle time.
(MPG)

Movie S5 Lateral view movie file generated from the best example using a 1.8 s gait cycle time.
(MPG)

Movie S6 Lateral view movie file generated from the best example using a 2.0 s gait cycle time.
(MPG)

Movie S7 Lateral view movie file generated from the best example using a 2.2 s gait cycle time.
(MPG)

Movie S8 Lateral view movie file generated from the best example using a 2.4 s gait cycle time.
(MPG)

Movie S9 Lateral view movie file generated from the best example using a 2.6 s gait cycle time.
(MPG)

Movie S10 Lateral view movie file generated from the best example using a 2.8 s gait cycle time.
(MPG)

Movie S11 Lateral view movie file generated from the best example using a 3.0 s gait cycle time.
(MPG)

Movie S12 Lateral view movie file generated from the best example using a 3.2 s gait cycle time.
(MPG)

Movie S13 Lateral view movie file generated from the best example using a 3.4 s gait cycle time.
(MPG)

Movie S14 Lateral view movie file generated from the best example using a 3.6 s gait cycle time.
(MPG)

Movie S15 Lateral view movie file generated from the best example using a 3.8 s gait cycle time.
(MPG)

Movie S16 Lateral view movie file generated from the best example using a 4.0 s gait cycle time.
(MPG)

Data File S1 GaitSym model specification file used to generate Movie S1.
(XML)

Data File S2 GaitSym model specification file used to generate Movie S2.
(XML)

Data File S3 GaitSym model specification file used to generate Movie S3.
(XML)

Data File S4 GaitSym model specification file used to generate Movie S4.
(XML)

Data File S5 GaitSym model specification file used to generate Movie S5.
(XML)

Data File S6 GaitSym model specification file used to generate Movie S6.
(XML)

Data File S7 GaitSym model specification file used to generate Movie S7.
(XML)

Data File S8 GaitSym model specification file used to generate Movie S8.
(XML)

Data File S9 GaitSym model specification file used to generate Movie S9.
(XML)

Data File S10 GaitSym model specification file used to generate Movie S10.
(XML)

Data File S11 GaitSym model specification file used to generate Movie S11.
(XML)

Data File S12 GaitSym model specification file used to generate Movie S12.
(XML)

Data File S13 GaitSym model specification file used to generate Movie S13.
(XML)

Data File S14 GaitSym model specification file used to generate Movie S14.
(XML)

Data File S15 GaitSym model specification file used to generate Movie S15.
(XML)

Data File S16 GaitSym model specification file used to generate Movie S16.
(XML)
